# Looking back at resignation syndrome: the rise and fall of a culture-bound endemic

**DOI:** 10.1186/s13010-025-00209-8

**Published:** 2025-11-28

**Authors:** Karl Sallin

**Affiliations:** 1 Uppsala University, Uppsala, Sweden; 2 Karolinska Institutet, Stockholm, Sweden

## Abstract

Resignation syndrome was endemic to Sweden for more than two decades. This paper explores how it emerged, sustained, and disappeared. Conceived to be caused by trauma and the stress of migration, permanent residency was considered necessary for recovery and became part of treatment. However, respecting national borders, affecting only some migrant groups, and with child abuse and feigned illness to gain asylum exposed, the understanding of the endemic shifted and aspects other than trauma and stress were acknowledged. Migration policy was altered with permanent residency permits replaced by temporary, and in legal procedures for residency, little weight was awarded to resignation syndrome. Management came to centre around child protection and sometimes involved separation of patient and family. The asylum procedure was kept separate from treatment. The strategy was found effective. Invoking residency as a part of treatment was suggested to have generated simulation but also expectations in turn producing and maintaining symptoms. In effect, as the illness-driving narrative and the incentivising medicolegal practice were reversed, the endemic faded. The thesis here put forward is that resignation syndrome was a condition created, maintained, and reversed by society. However, it is not the first, nor is it the last. Trending illness manifestations in general may benefit from a constructivist analysis, the resignation syndrome example showcases how, and that mitigation is possible.

## Introduction

This paper recounts the rise and fall of resignation syndrome. The objective is to explain how it emerged, sustained and disappeared in order to elucidate the phenomenon, but also offer a case report of a culture-bound endemic from which we can learn.

The analysis draws on scientific and other publications, such as clinical guidelines, transcripts of hearings, governmental official reports, newspaper articles, books, etcetera. The condition’s puzzling, perhaps even mysterious [1], properties including loss of function without objective findings, the rapid rise and fall, geographical distribution, and selective affliction, will be examined in a synthesis employing a constructivist understanding permitting social and cultural factors to explain nothing less than what has been considered a medical condition.

The debate between realists and antirealists, endorsing reality to exist independently or to be constructed through discourse, respectively, is fundamental [2, 3]. Following ‘the language turn’ in philosophy, and the works of Foucault, in which reality including mental illness was reduced to discourse altogether [4], later versions of constructivism have generally been less extreme [5–7]. One influential account employs the notions “natural kinds” and “interactive kinds” with the former taken to exist independently of our classification systems encompassing for instance entities of physics, and the latter shaped by social and scientific discourse with mental illness categories prime examples [7]. Integrating aspects of realism and antirealism, this moderate version suffices for the present purposes. It supplies the orthodox realist understanding of medical disorders with an alternative permitting sociocultural cues to affect, even generate illness. Interestingly, in contemporary cognitive neuroscience, “priors”, expectations coded in brain structure and function, which permit bottom-up and top-down determination of percepts and cognition, are employed and presumably accommodate realist and antirealist notions simultaneously [8–10]. In a coming paper, this hypothesis, which would support an understanding incorporating biological, psychological, and sociological levels of analysis, and their interdependency will be examined. For now, however, constructivism as an alternative to realism offers a way to make certain phenomena engaged by medicine, such as resignation syndrome, intelligible. Through the illustration of how context and discourse can impinge on and create illness, an understanding of similar–present and future–phenomena could start to emerge. Thereby medicine and society could become better equipped and perhaps able to prevent and mitigate culture-bound endemics.

## Background

**Fig. 1:**
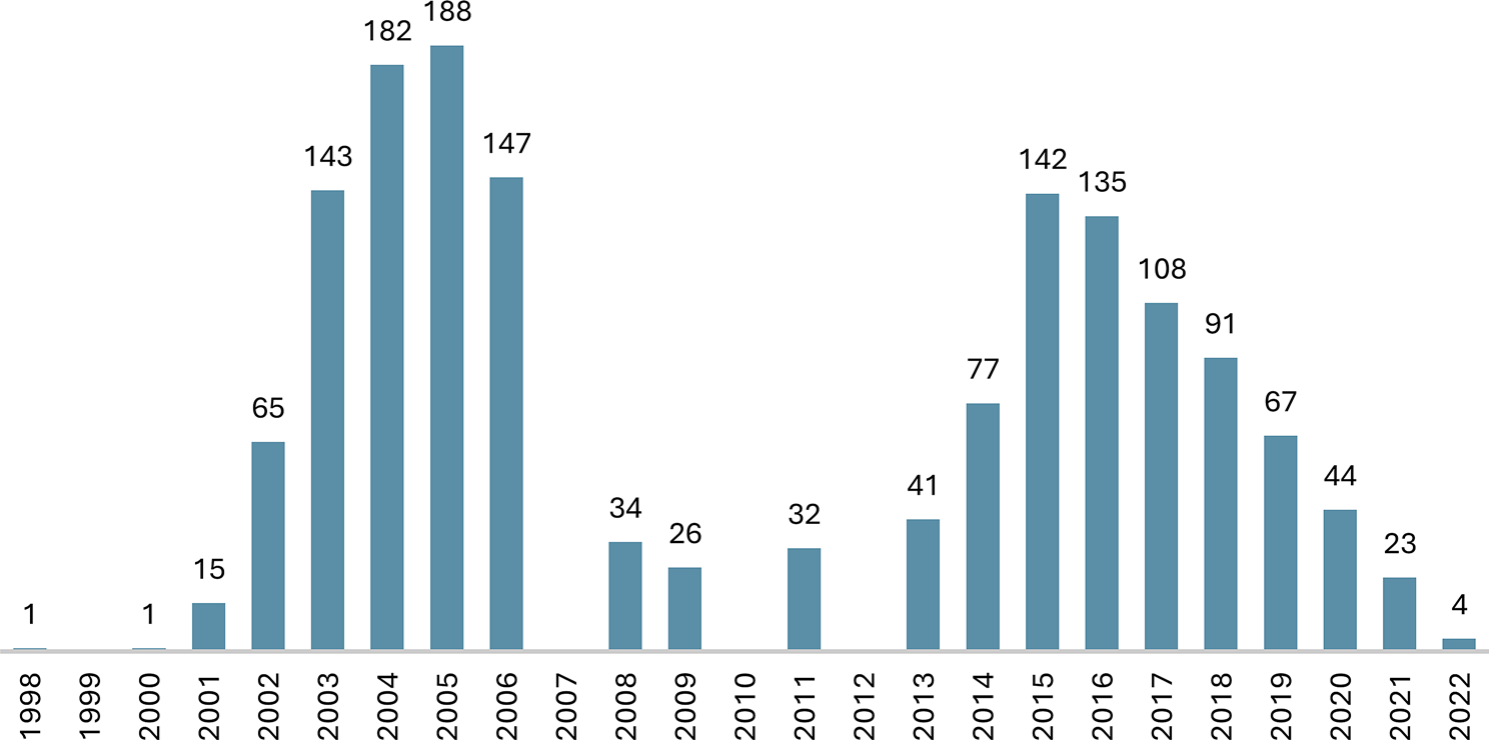
Reported cases of Resignation syndrome 1998-June 2022. In 2014, resignation syndrome (previously apathy) was recognised by the Swedish National Board of Health and Welfare and awarded a national ICD-10 code (F32.3 A) to enable statistics. Prior to 2014, data is of varying quality and collected neither systematically nor regularly. In 2006 a general amnesty was implemented after which surveys became less frequent. Sources [11, 12, 34, 50–52]

For more than two decades, resignation syndrome, characterised by profound behavioural change approximating a coma state, afflicted asylum seekers in Sweden. Trauma and stress were endorsed as causal, and asylum as the cure [11]. Restricted to migrants from particular regions, respecting national borders, and with incidence rising and falling rapidly (Fig. 1) the label *endemic* is warranted. Today, with prevalence near zero [12], it has passed, and its examination is due.

Trending medical disorders have been subject to analysis integrating social and cultural rather than biological factors [13–15]. Legitimisation by authority–diagnostic labelling, typically by medical experts–is incentivising per se but may also sanction relief from obligation and award external gain by unlocking benefits and welfare resources, and it has been invoked to account for why conditions evolve and resolve in space and time [14, 15].

However, according to the predominant understanding in medicine, a medical disorder is exhausted by objectively assessable variations from biological norms reducing survival, reproductive, or other function. This entails that disorders are processes of nature which afflict and transpire regardless of norms and practices. On this biomedical model it can be difficult to explain how sociocultural factors can affect the prevalence of a condition, and even more so, how novel conditions can thus emerge. As a result, entities exhibiting endemic properties are instead often rejected. Conversely, if recognised as illnesses, thus lacking objective evidence and merely experiential, they may be attributed to imagination, madness, or simulation. Counterintuitive to the scientific ideal to which medicine aspires, and the biomedical model of disorder, they are then furthermore awarded low status with negative impact on patients [16, 17]. It is not only that the biomedical model is insufficient with regards to trending medical disorders. Also, the lack of an alternative other than subjective illness may cause harm.

A constructivist understanding appears better suited for the analysis of trending illness. On this conception, medicine as science and practice rather than uncovering an inert landscape of disorders, affects and, on a strict interpretation, creates it [18]. Disorders then, are products of social construction *and* science, with the latter perceived to be a social practice [18], with the scientist an actor partaking in a plot under construction. Importantly, on moderate versions of constructivism, neither the ontological claim that natural entities exist, nor that science is a meaningful enterprise serving their characterisation, needs to be rejected [19]. Instead, the constructivist stance can be regarded as a complement to the biomedical model [7, 19].

One upshot of this understanding is that medical conditions can be subject to negotiation. Those affected by a disorder, the physicians labelling it, the media exposing it, the industry profiting from it, the insurance system awarding the sick-role, and all other entities which give it meaning contribute [13–15, 18, 19]. With stakeholders providing different perspectives, conditions are presumably shaped accordingly. Thus, they acquire and rely on characteristics reflecting an amalgam of factors inherent to their sociocultural context, and consequently, as this context is dynamic, expressions may arise, change, and disappear [14, 15]. A varying panorama of disorders can thereby be accommodated.

Furthermore, the normative capacity integral to the biomedical model ensuring moral neutrality is revoked when conditions are no longer perceived to exist and affect independently of observer and context. Instead, nonnatural norm-setting is entailed, and diagnoses become laden with values reflecting different perspectives [18, 20]. When affected by diverse pressures and exerting diverse pressures [21], medical conditions may become instruments of power [4, 18]. Politics, advocacy, business, and other interests are invited and enforce their agendas. Awarding the sick-role to unlock treatment and benefits, but also disciplining citizens into obedient workers, addressing social and other inequalities, and creating consumers of health-related products and healthcare, impact what meanings medical conditions are awarded, including their moral connotations, and thereby also determine their appeal to patients and society. Consequently, diagnoses serve not only as descriptions but also as templates, with variable attraction, for how illbeing is understood and expressed [22].

Perhaps the most influential stakeholder is medicine itself as it embodies the perspective of objective science which status is unprecedented. Indeed, the importance of diagnostic labelling has been underscored and endorsement of diagnoses argued to promote trending illness [14, 15]. It is important to note that labelling affects self-perception, and behaviour, and thereby may give rise to illness and in effect cause harm beyond what is integral to the underlying condition–if there is one [18]. Entities like fibromyalgia, chronic fatigue syndrome, and gender dysphoria, to name only a few, have become recognised although their validity has been put into question [23–25]. To what extent these and other related labels conceal and create problems rather than solve them has been debated [18, 26]. The distinction between prudent and iatrogenic use of diagnoses is not given but rather bound to emerge as medicine is practiced.

The merits of the constructivist approach to the analysis of trending medical conditions, which seem to rely more on sociocultural than biological factors, are tangible. Nevertheless, in mainstream medicine it appears underexploited. In the DSM-5 it is asserted that all mental disorders are shaped by cultural factors [27]. Yet, the same taxonomy emphasises a phenomenological approach refraining from invoking causal factors. Perhaps this attitude prevents sufficient recognition of important factors on the sociocultural level, and thereby renders a, in other aspects heterogenous, set of conditions, invisible. If this conjecture is correct, a unitary understanding could facilitate recognition and understanding of what arguably is a serious, and partly self-imposed [28], challenge to medicine and society [29]. Highly controversial phenomena such as myalgic encephalomyelitis/chronic fatigue syndrome and rapid onset gender dysphoria, but also attention deficit hyperactivity disorder have indeed been suggested to benefit from an analysis invoking sociocultural factors [30–32].

Trending medical conditions, including culture-bound endemics, thus warrant attention [29], and individual entities provide important data points [33] which when added up can further a joint analysis. Resignation syndrome is here showcased as one of such data point [22, 34] which can supply insight of value in countering similar contemporary or future phenomena.

## Trauma and stress, and the rise of an endemic

The first known case of what later was labelled resignation syndrome dates from 1998. A 17-year-old boy with Chechen descent arrived in Sweden with his family. Reportedly, he had suffered abuse in Murmansk, Russia, where the family initially had sought asylum from the first Chechen war. In Sweden residency was denied, and he deteriorated into “apathy”. Nine months later he recovered after a reversed decision [34].

The case is typical. Children and adolescents belonging to minorities from former Soviet republics and former Yugoslavia were overrepresented [34–42], and assertions of assault, rape, or murder in the close family common [39, 42, 43]. A negative event such as an adverse asylum decision often predated presentation [42–44]. Unspecific depression-like symptoms, stupor and eventually unresponsiveness prompted a generally unremarkable workup, and admission [35, 40, 44]. Electroencephalography and sleep behaviour demonstrated normal sleep wakefulness cycles [35]. Clinical observations suggested awareness to be intermittently preserved, at least in some cases [45]. Despite long periods of unresponsiveness, and tube-feeding lasting for months or years [34, 39–41, 46], restoration of function appeared complete [41, 47] with rare exceptions [48, 49].

In the beginning of the millennium, numbers rose rapidly, see Fig. 1. Prior to 2014, when data was sporadically collected using different methods, it is estimated that more than a thousand minors were afflicted [22] and from 2014 to 2021, the National Patient Register holds 432 cases [12].

From the outset, debates over how this phenomenon should be understood and confronted roared. At a seminar hosted by prominent paediatricians a kind withdrawal reaction was suggested:

*The condition could probably be described as a “give-up syndrome”*,* and the children show similarities to those who were called “Musselmänner”. Musselmänner were the concentration camp inmates who had completely lost the will to survive and who withdrew from the outside world in a manner similar to autism. Apathy reactions as an expression of “learned helplessness” have been observed in animal experiments when animals are exposed to unpleasant stimuli from which they can neither fight nor escape.* [53]

An early report on “depressive devitalisation” from 2004 [54] came to exert great influence [53, 55, 56]. Novel to refugees, it was asserted that the condition otherwise overlapped with *pervasive refusal syndrome*, and somatisation, allegedly due to pathological interplay between patient sensitivity and parental hopelessness and helplessness, was invoked. In-patient care and tube-feeding were claimed necessary until relief by permanent residency could be achieved. Predicaments of asylum seeking, including insecure living conditions and repeated negative decisions, as well as unprocessed trauma, and underlying mental vulnerability, were suggested causal [54].

Consequently, in clinical practice, previous trauma and stress of living under the threat of expulsion were recognised as precipitating [11, 57]. Securing a safe environment was therefore considered crucial and permanent residency invoked as a therapeutic intervention [11, 57]. The family, in paediatric practice the primary resource in stress and trauma treatment, as well as target for intervention, was relied on to supply hope and reassurance, and furthermore entrusted with nursing, maintenance of a daily routine, and sensory stimulation [11, 57], although the outpatient regime was debated with some favouring admission [53]. With residency pending, interventions were however in effect often considered futile [11] and lack of recovery excused. In a national guideline it was noted that:

*Among therapists with direct experience of caring for these families*,* permanent residency is considered by far the most effective “treatment”. No treatment feels urgent to the families until it begins to become clear to them that something is happening in the residency permit issue itself. (p48)* [11]

When residency was awarded, the condition nevertheless often persisted unchanged for many months [40].

The phenomenon generated considerable public attention and prompted comprehensive official and expert reports [37, 38, 50, 51]. A range of hypotheses including biomedical models, family psychology system theory, psychological factors including trauma, political factors related to the asylum process, culturally inherent or generated idioms of distress, and intentional accounts including Munchhausen by proxy were suggested.

However, when suspicion of manipulation arose, categorical dismissal [53, 58–60] and support [61–63], neither at the time well substantiated, ensued. Involving exposed children, the debate exploded [58, 59, 64] resulting in increased polarisation [65–73].

With residency contingent on the risk of repercussions upon returning to the mother country, an incentive to assert previous violence and trauma was recognised [50, 51], and wanting evidence such narratives were questioned [63]. One critical commentator noted several peculiar circumstances:

*Firstly*,* it is strange that the prevalence in Sweden has increased from zero to more than four hundred in just a few years. Even though one can find isolated cases with similar symptoms in the literature*,* it is obvious that the explosive spread must be due to some previously unknown factor.*

*Secondly*,* it is strange that the condition almost exclusively affects children in refugee families where the question of asylum has not yet been decided or has been decided to the family’s disadvantage. This fact suggests that the condition is directly linked to the asylum process itself.*

*Thirdly*,* it is strange that only refugee children who live with their parents*,* but not refugee children who have come to Sweden alone*,* have been affected. This fact suggests that the presence of the parents is a prerequisite for the onset of the condition.*

*Fourthly*,* it is strange that the affected children largely only come from states in the former Soviet Union or states in the former Yugoslavia. This suggests that the condition is in some way connected to these states.*

*Fifth*,* it is strange that the condition*,* if it were to be caused by psychological stress*,* does not affect children who have been exposed to other forms of such stress than the very stresses that an asylum process can entail.*

And concluded that:

*A combination of these peculiarities raises the suspicion that the condition is the result of some form of manipulation*,* aimed at increasing the chances of obtaining a residence permit. (p856-7)* [63]

In summary, the surging prevalence, debut while awaiting, or after a negative asylum decision, selective affliction however sparing unaccompanied minors, and other forms of stress failing to provoke debut, elicited suspicion of intoxication of children, or others coerced to pose as such [63]. Also, simulation [61] and group malingering by proxy [62] were suggested. Anonymous reports [74], therapists’ experience [64], and accounts of Russian “travel agents” offering advice including what drugs could induce symptoms [75] were mounted in support. Iatrogenic [76], cultural [38], or familial [37, 38] factors, and Munchausen by proxy [35] were also proposed but received less attention.

In general, feigned symptoms were rejected by avid clinicians [53, 55, 56]. Proceedings from a seminar organised by the The Swedish Paediatric Society’s Working Group for Refugee Children summarised the conviction thus: “There is consensus that manipulation and poisoning can be dismissed as explanations for the accumulation of children with resignation syndrome” [55].

Although confirmed [77], even officially recognised [50, 78], feigning nevertheless did not stir this conviction. Instead, the trauma and stress conception seems to have been reinforced. At an expert hearing organised by the National Board of Health and Welfare [56], pervasive refusal syndrome, unrecognised in official taxonomies, and based on a small number of case reports of non-migrant patients exhibiting *active* refusal [79, 80], was still the favoured label, whereas Munchausen by proxy and conversion syndrome were rejected. The hearing maintained that

*It is […] somewhat shocking that claims of parentally induced illness have been able to emerge so easily and have been so little questioned […] There have even been accusations that parents have directly poisoned their children […] The severity of the symptoms alone should be enough to refute such claims. Children do not take direction like that! (p9)*

And, invoking an unconventional interpretation of conversion disorder and, moreover, in any case, gratuitously assuming absence of internal psychological conflict, that

*The common denominator in conversion syndrome is thus a specific*,* predisposing personality together with complex internal psychological conflicts – not psychological trauma of an observable kind. This means that basic clinical and epidemiological data regarding refugee children with pervasive refusal syndrome do not fit the picture of conversion syndrome. (p9)*

Assuming previous trauma [53, 55], indeed commonly reported [43, 54] yet challenged [63] also by evidence [39], and with debut [39, 46, 54] or recovery [46, 54] coinciding with residency decisions, an understanding attributing previous and renewed traumata, and the stress of living under threat of expulsion was authorised [53, 55, 56]. Regional distribution and selective affliction [39, 43, 46, 54, 76] failed to disturb this conception. Instead, personal accounts, unpublished reports [56], and historical behavioural patterns [81] were invoked to relativise these findings, and speculations over unbearable insecurity pertaining to the asylum process as well as reduced parental capacity, and insufficient societal and official support were advanced [56]. Theoretic models invoking stress were proposed [57, 82]. Studies of cortisol levels [43] and steroid metabolites [83] were despite inadequate designs interpreted in support, and erroneously argued to exclude manipulation [84]. Negative drug screenings [40] were considered to disprove intoxication [55] despite inadequate methodology [59]. As a result, the perception of resignation syndrome as a genuine trauma and stress induced disorder prevailed [43, 53, 55, 56, 85].

Additionally, moral arguments were raised. *The Declaration of Lisbon*, stipulating a duty to secure treatment, also when opposed by legislation or official bodies, was invoked [53, 56, 86]. Suggested political action [55] absent, civil disobedience was argued a duty [86, 87], and the human rights perspective an obligation [88] which could be honoured through medical certificates. The chief physician of Region Skåne, a large municipality in the south of Sweden, urged

*every doctor to continue to protest against the deportation of apathetic refugee children. Primarily for the sake of the children*,* but also for our own sake*,* that of the doctors*,* so that the ethical mission that medical ethics dictate*,* and that the outside world probably expects of us*,* can be fulfilled. (p1961)* [87]

*Humanitarian grounds*, a malleable legal requisite [58, 59] accommodating medical need, was invoked to achieve permanent residency in the face of deportation, and with appropriate medical care asserted inexistent in the receiving countries, expulsion was argued not only inhumane but life-threatening. This practice was motivated through moral arguments, yet at the same time it was promoted as a medical treatment [11]. However, relevant retrospective studies [40, 41, 46] were not part of the documentation, and that former patients awarded residency little importance for recovery [48] received no notice.

The debate over genuineness was inexhaustible. The idea that families could stage a medical condition, thus use their children, to increase chances of residency was vehemently rejected [53, 55, 56, 67]. In appreciation of symptom reversal in six cases ensuing separation of family and patient [59], the National Board of Health and Welfare articulated the need to consider manipulation in the workup [78]. However, The Swedish Paediatric Society’s Working Group for Refugee Children expressed “shock” over the miscrediting of a vulnerable group and urged for a review omitting any mention of manipulation [67]. In parallel, a prestigious investigative TV program–later in a highly unusual self-audit characterised as “100 per cent on one side in the opinion war” [60]–declared there to be no proof of manipulation [38]. The memorandum was subsequently retracted [59].

Journalists have later defended suppressing evidence of manipulation by appealing to the possible harm it would have brought onto an already vulnerable group [58], a fear shared by some doctors [67]. Speculatively, this reaction resulted also from an unwillingness to acknowledge that migrants, like people in general, could engage in reprehensible activities under certain circumstances, and that those circumstances were present in Sweden. Conceding as much would have played into the hands of immigration sceptics and was therefore presumably not an option. The rejection of an official report suggesting manipulation to be one of many factors which jointly or individually could account for cases of resignation syndrome [38], and the public vilification of its author [58, 59], can be interpreted from this perspective. The focus on trauma and stress [58–60] served to distance the debate from manipulation and, although there were exceptions [63, 74, 75, 89], promoted a biased narrative [58–60].

With public attention massive and the phenomenon implicating matters of immigration policy, politicians had to–or willingly engaged. To some, the apathic children became or was made into a political asset [58, 59]. The Easter Appeal of 2005, led by the Church of Sweden, supported by non-governmental organisations and political parties, demanded asylum for patients and their families. A representative from Folkpartiet (a social-liberal party) claimed that

*Children who have entered a state of depressive devitalization*,* the so-called apathetic refugee children*,* are in an extremely serious situation. It is a shame for Sweden that we cannot give these children humane and dignified treatment. I therefore welcome the fact that the Immigration Board is now recommending that the government allow these sick children to stay in Sweden.* [90]

The Easter Appeal resulted in a near government crisis, avoided only by the passing of legislation in effect granting residency to most asylum-seeking families with children, *regardless* of medical need, in 2006 [58]. The force evidently unleashed drew political attention also to the nature of suffering resignation syndrome involved, and criticism of the prevailing narrative provided an opportunity for the defamation of political opponents [58].

A conception at odds with evidence in official reports [38, 50, 51] and that of at least some clinicians [59, 60, 76] had become the official narrative. Together with a fear of stirring antiimmigration sentiment, it would block the debate for years to come. Indeed, despite knowledge, albeit anecdotal, of separation to have enabled recovery and manipulation to have occurred, the National Board of Health and Welfare in 2013 again neglected mention in a national guideline [11], which ironically however apostrophised a children’s rights perspective [59, 77].

In 2014, resignation syndrome, and a national ICD-10 code (F32.3 A) were officially recognised to enable epidemiological studies [11]. *Resignation* implies an aetiology, and *syndrome* appoints its remedying to medicine. This firmly established the condition and its aetiological understanding within, and perhaps beyond, the Swedish medical community.

The polarised debate in which resignation syndrome was conceived, has been suggested to reflect pre-existing sentiment [59] which arguably served the emergence of an illness narrative and a medicolegal practice. Immigration was controversial in Sweden throughout the 1990s following an increase in the previous decade. A narrative of allegedly defenceless migrants denied residency by evil officials was exploited by media [58] even receiving a newsroom label of its own (*Expressenmallen*) [59, 91]. These campaigns for residency pleading humanitarian grounds often highlighted cases involving children [58]. Polarisation was further fomented in the media when allegedly xenophobic little people were contrasted to pillars of society engaged in the fight against racism [91]. That false information, fake identities, and marriages of convenience were used to circumnavigate the legal process, and that medical conditions were exaggerated and imposed to satisfy the humanitarian grounds requisite, and that doctors received and met requests from patients and activists to write certificates tailored to those grounds, received little attention [58, 59]. Considering this backdrop, when the first case presented in 1998, sentiment, norms, practices, and institutions which could serve to reinforce symptoms and drive an endemic have been argued to already have been in place [58, 59].

In recognition of the circumstances and chain of events here now recounted, an interpretation invoking sociocultural factors to explain the emergence and continuation of the resignation syndrome endemic is plausible. Pre-existent polarised sentiment, norms, practices, and institutions–particularly activistic media and a malleable legal requisite–conceivably provided auspicious circumstances. Involving children, and with the debate orchestrated by the media and activists, a biased narrative tailored to receive maximal public attention was endorsed, and interest grew rapidly. Trauma, stress, uncontrollability, helplessness, and hopelessness recognised as causal, the model illness of pervasive refusal syndrome, relativisation of endemic qualities, proposed theoretic models, biased interpretation of research data, an official diagnosis, and expert backing appears to have contributed to the emergence and solidification of an illness narrative. Moreover, with permanent residency the recommended remedy, a medicolegal practice emerged and conceivably, together with the illness narrative, vested the behavioural pattern with a capacity for endemic spread.

Furthermore, with doctors’ default empathetic attitude and predisposition to protect the rights and lives of presumably innocent minors, moral obligation compelled action, including resistance to report manipulation, activistic use of medical certificates, and endorsement of unevidenced treatment. On the political level, an appeal to human rights and the fear of arousing antiimmigration sentiment directed and controlled narrative and actions. With children and families attaining status as political assets, and acceptance of the illness narrative signalling compassion and humanism, refutation was deterred by the reprehension it risked eliciting. Consequently, pushed from media, politics, doctors, and activists, one reinforcing the other, the illness narrative became incontestable.

Moreover, crucially, the apathic children became a symbol serving values and objectives already embodied in Swedish society [58–60]. Concern over reduced political influence *as a result of recovery*, was even voiced by a board member of an NGO. In this sense, not only were the patients and families relying on society’s sanction. Also, they were instrumental in the promotion of agendas parts of society already sought to realise. Without this reciprocal relation, the resignation syndrome endemic appears difficult to envision.

## Epidemiological inconsistencies and the fall of an endemic

In 2016, a paper (again) noted the regional distribution of resignation syndrome to resist explanation on a trauma and stress hypothesis. Instead, it was proposed that the condition could be understood by invoking the concept of a *culture-bound condition* [22]. The argument was that since trauma and stress are pervasive in migrant populations, hypothetically if they are causal resignation syndrome should be widespread, however with the endemic restricted to Sweden trauma and stress are insufficient, and contextual factors implied.

Coincidentally, also in 2016, Swedish immigration policy was tightened after an increase of asylum seekers in the preceding years. Resignation syndrome ceased to constitute a *particularly difficult circumstance* (heir to the humanitarian grounds requisite lowering the bar for asylum) and as a result, impact on the asylum procedure diminished. Moreover, the policy of awarding permanent residency was discontinued in favour of temporary permits of thirteen months [52].

In 2016, the incidence started falling and the trend has so persisted, see Fig. 1. Only twelve new cases were reported in 2020, six in 2021, and in 2022, one to three–as compared to 62 in 2017 [12]. The rate of decline exceeds that of asylum seekers to Sweden in general–down 34 per cent from 2017 to 2022, and that of asylum seekers aged six to 17 from countries from where resignation syndrome cases typically have originated (former Soviet countries, former Yugoslavia, and Turkey)–down 54 per cent [92]. Thus, reduced number of asylum seekers does not appear to account for the decreased incidence. However, apart from the policy changes, and the criticism of the trauma and stress account, also other shifts and events likely contributed.

In 2019, malingering or malingering by proxy, including abuse [77], enacted to gain asylum [34, 59, 93, 94], were exposed and reignited the debate attracting former champions and new challengers engaging in yet another set of fierce encounters laying bare the polarised positions. This time, however, former patients, now victims of child abuse, and their stories, had become public:

*I feel so let down by the healthcare system*,* even by the kind nurses who came to my house every Monday. Get out of your bubble! It’s us kids who get stuck. We’re the ones who get all the crap because no one can think a step further. No one asks us how we’re doing. Everyone talks to us through our parents. (p69)* [77]

*Nermin was convinced that all the nurses*,* doctors and specialists knew deep down that he wasn’t really sick. But no one said anything. No one told his parents to get real. Not even when it was obvious that they were trying to make him lose weight. His only function was to give the family a residence permit. But how long would it take: a year*,* two years*,* three years?*

*Nermin looked around the small boy’s room that had been his prison for twelve months. There were no sharp objects there. Nor was there any point in jumping out of the window since they lived on the second floor. But he had a bunk bed and a long cord that ran from his PlayStation console to the wall outlet. (p48)* [77]

Rejection of simulation and abuse was in light of such testimonies no longer possible, and instead the official narrative started to disintegrate.

Also, the treatment principles relying on family-involvement and permanent residency endorsed in a national guideline [11] were by the Swedish Agency for Health Technology Assessment and Assessment of Social Services (SBU) demonstrated to lack scientific support [95]: “After a literature search, SBU’s information service has identified neither any relevant systematic review nor any relevant primary study on the reliability of diagnostics or effects of treatment for resignation syndrome” [95]. Simultaneously however, it was discovered that in one small cohort, the reversed practice–separating patient from the rest of the family, and abstaining from invoking residency in treatment, promoted recovery, while standard treatment exhibited no positive effect [34, 96]. The suggestion of an iatrogenic or sociogenic endemic was made [34], again [76]. The trauma and stress narrative, and the practice of supplying the legal system with medical certificates endorsing recovery to be dependent on residency, were proposed to drive symptoms, and malingering. In essence, the medicolegal practice and the curated illness narrative were suggested to explain the regional distribution [34, 97].

To facilitate professional discussions and possibly a reorientation, starting in 2019, the National Board of Health and Welfare housed a series of meetings. Incidence and prevalence updates were communicated, and although discussions initially were tense, the illness narrative was gradually abandoned, and the possibility of the medicolegal practice partially having curated the endemic was recognised. Eventually, the national guideline [11] emphasising the importance of residency was retracted. With the Board operating under new instructions and no longer enjoying the entitlement of recommendation issuance, there was no official replacement. Separation, refraining from including residency in treatment, and vigilance towards simulation were however endorsed in a policy brief from Uppsala University [98] which also summarised the worry that resignation syndrome was a result of policy and practice:

*Regarding resignation syndrome*,* based on the results we have reported*,* it is necessary to ask whether the understanding of the condition as dependent on stress and trauma*,* which is remedied in particular by the issuance of a residence permit*,* in fact has given rise to the accumulation of cases of the condition in Sweden. Through a certain understanding*,* the condition has been legitimized and through a certain treatment*,* an incentive has been created which has made resignation syndrome relatively common in Sweden. (p2-3)*

With previous management principles discarded, the medicolegal practice–presumably having served to incentivise resignation syndrome [34, 76]–was inverted. It appears the rejection of resignation syndrome merely as trauma and stress-induced resulted in a change in mindset among healthcare professionals. The default trusting attitude relaxed, and clinicians instead urged first and foremost for a child protection perspective [94, 98, 99] enabling the release from the arguably illness driving narrative asserting residency to be necessary for recovery. With regards to treatment offered without invoking pleas for residency, a paediatrician noted:

*There is now clinical experience of how we can help the children. The children’s clinic in Falun has successfully changed its approach. Short-term separation procedures have prevented new cases of illness*,* and rigorous rehabilitation of the families has led to the children regaining their functions. Since the new approach was introduced*,* all children have recovered*,* with the exception of one family that moved from the region.* [99]

In parallel, as feigned illness and social contagion was acknowledged, frustration and reprehension grew, along with calls for officials to assume responsibility [100]. The change in sentiment is reflected in the response given by doctor Göran Bodegård–author of key publications [35, 46, 54], feverish advocate for the apathic children, and the most conspicuous professional figure throughout the endemic–to the question “[d]o you think manipulation or Munchausen by proxy could explain why so many were afflicted?”: “Yes, absolutely” [59].

## Reiteration and reinforcement

The conception of resignation syndrome chiefly as trauma and stress-induced lingers still today [42, 101]. The condition has been popularised on film [102, 103], and in media reports children labelled with resignation syndrome still fit right in [104]. In the scientific literature, the distinction from other conditions is not always made [105]. However, with case load demonstrated to correlate to changes in immigration policy, treatment and management, and other external circumstances, the evidence for a discrete contextually dependent entity is strong. Nevertheless, a few more remarks, in part offered previously however only in Swedish [97], add to the evidence.

The trauma and stress hypothesis fails to align with epidemiological observations. In 2014, 5.1 per cent of asylum-seeking minors under psychiatric care in Sweden suffered from resignation syndrome [106]. Yet, as previously noted [22], an official report found no resignation syndrome cases in the countries from where the groups afflicted in Sweden migrate [38], which was unexpected considering that threats, persecution, and violence often were disclosed as motivations for migration [43] and would have been expected to generate symptoms regardless of location. Moreover, despite increasing international attention [1, 22, 102, 107–109], limiting the risk of underreporting, cases outside of Sweden remain rare (and disputed) which is surprising considering the omnipresence of trauma and stress in migrant populations worldwide, including other European countries which received migrants from the same countries as Sweden.

Similarities to pervasive refusal syndrome [44], another label unrecognised in international taxonomies [110], have been taken to support the claim that resignation syndrome is not restricted to Sweden [82, 105, 111]. However, in contrast to resignation syndrome, pervasive refusal syndrome, as originally described, occurs sporadically in resident majorities [50] and differ in clinical gestalt [22, 36]. Furthermore, cases labelled resignation syndrome [112] or pervasive refusal syndrome, have been reported from a migration facility in Nauru [113, 114]. Involving hunger strikes and self-harm, these differed from resignation syndrome (see [22] for list of reported symptoms, and supplements to [34] for diagnostic proposals). However, even acknowledging disputed cases, and regardless of label, resignation syndrome was endemic to Sweden, and nowhere else, in contrast to what is predicted on a trauma and stress account.

Moreover, not only did resignation syndrome respect national borders. Also, it afflicted selectively certain migrant groups [34, 36, 38–42, 44], a finding which seems not to be explained by traumatic burden [50]. Other migrant groups, having asserted comparable traumata, and subjected to equal stress-inducing contextual factors through co-location and similar predicaments, nevertheless resisted affliction [39, 59, 76]. A history of trauma is not even–in fact far from–a consistent finding [34, 39, 40, 76]. Thus, neither traumatic burden nor levels of stress distinguished the afflicted group. This finding, of course, does not contradict that trauma and stress were prevalent among those afflicted nor that they played a role in resignation syndrome. It does however show that neither are sufficient, and with regards to trauma, not even necessary, to the inception of resignation syndrome.

An illness narrative and a medicolegal practice can explain the regional distribution of resignation syndrome. Data, although limited, can be taken to suggest the sociocultural analysis to apply also to the selective affliction.

A restricted endorsement of the illness narrative and knowledge of the medicolegal practice within subgroups could explain the selective affliction. Reports from Gällivare, Stockholm and Sundsvall [59] support this suggestion, and a victim of malingering by proxy stated that the modus operandi of using the diagnosis to obtain residency had been a well-kept secret among her countrymen. Also, organised illegal trafficking of migrants from certain countries was reported and involving intoxication [75] it could account for cases of malingering or malingering by proxy. Conceivably, an illness model, internalised and exhibited in genuine, presumably functional, presentations [115], could have been born out of such malingered cases.

Indications of culturally specific help-seeking behaviours adhering to the afflicted groups can, furthermore, be traced. A particular expectation involving the doctor to do the remedying, and reciprocal passivity on the family’s behalf, were observed and suggested to correspond to a culturally specific help-seeking behaviour exhibited by families from former Soviet countries [76]. Similarly, a particular reaction [2, 41] involving reduced parental agency or even obstruction (“lethal mothering” [35]) as well as sympathetic behaviour (in some instances referred to as *folie à deux* [35]) including self-starvation, were noted in families from former Soviet countries. With some other migrant groups recognised to refrain from seeking medical assistance in general, allegedly due to lack of trust [76], differences in help-seeking frequency and behaviour may have been of importance.

The internal circulation and reinforcement of an illness narrative in a group, partly isolated, as in a new country, yet tightly knit together by common origin, language, or similar factors, and underlying culture-specific help-seeking behaviours, could presumably elicit a symptom pattern taking on endemic proportions provided the behaviour is incentivised. Such a behavioural pattern may have originated in an index case of malingering, or catatonia [22], the latter endorsed as a stress related neuropsychiatric reaction pattern and with symptoms partly overlapping those of resignation syndrome [116].

## Feigned and functional

Polarisation between those conceiving of resignation syndrome as a condition afflicting vulnerable minors the assistance of which is a moral obligation, and those asserting fraud or exploitation of innocent children to warrant punishment of, in any case, the parents, has been underwriting the endemic throughout its course. In theory, the distinction between simulation and involuntary symptoms is clear. In clinical practice there is however often an overlap which may run deeper than what can be explained by the epistemological difficulty of telling them apart in clinical evaluation [117].

Setting these intricacies aside, the polarised positions, which in themselves have prevented a dispassionate deliberation the lack of which arguably was decisive to the inception and maintenance of the endemic, are not helped by dearth of data. Alas, assuming a trauma and stress aetiology, and with manipulation dismissed “by consensus” [55], studies have been designed neither to establish simulation nor involutory symptoms. With treatment managed by the families and conducted on an out-patient basis [11], the possibility of coercion, unintended negative family effects, or tertiary gain has typically been neglected. Also, separating clinically often indistinguishable presentations such as malingering by proxy and functional neurologic symptom disorder, linked to trauma, stress, incentives, and familial factors [118], is notoriously hard. Data in support of feigned symptoms, coerced or not, as well as involuntary symptoms are thus scarce.

However, roughly 40 cases of malingering, malingering by proxy, or factitious disorder imposed on another, attested to by victim or perpetrator, or established based on circumstances offering no other explanation, have been reported. A journalist recounted approximately 30 cases [59, 77] involving parental coercion with or without force, including two cases of drug administration by caregiver, and others where intoxication to elicit symptoms was considered probable. Further, a newspaper article exposed two cases also awarded social benefits [93]. Three cases were discovered in a retrospective study [34], two in clinical practice [94], and this author has encountered three cases. Additionally, numerous cases in which feigned symptoms were suspected remain unassessed [119]. This author has on several occasions been contacted by the police regarding suspected simulation including drug-induced symptoms.

The discrepancy between history and objective findings, in particular a lack of muscle wasting despite months to years of asserted immobility, is remarkable and consistent with feigned illness, yet rarely acknowledged among commentators. Furthermore, in contrast to the consensual conviction of an authentic condition [55], a Master’s thesis conducted early in the endemic found all nine staff members interviewed to be convinced that although some patients were genuinely ill, the majority purposefully exhibited symptoms [64]. Moreover, a doctor recently shared his experience of 40–50 adult patients which he claimed malingered apathy in 2004 to 2006 [119]. He also claimed his insights were rejected by peers leading to self-censorship [119]. This accords with accounts given today by nurses and nurse assistants previously engaged with children diagnosed with apathy. In confidence they express that when having tried to convey what they considered to be evidence of feigned illness, this information was often suppressed. Conceivably, doctors dismissed their observations or attributed them to variable symptoms supposedly inherent to the condition as such, seemingly counter to the obvious explanation but coherent with the physician’s dictum to always trust the patient, or perhaps reflecting a moral conviction commanding leniency in order not to disrupt the possibility of a positive asylum decision [53, 56, 87], or simply due to the uneasiness attached to serving as an inquisitor and condemner [120]. Balancing different ethe–do no harm and remain truthful–integral to the role of the physician in this situation, poses a dilemma. The power to absolve or condemn confers an unsolicited role which has been chartered in another context strikingly similar to that here discussed [121].

Undoubtedly, simulated illness explains the resignation syndrome endemic, at least in part. However, psychiatric symptoms prior to debut [34, 40–42, 44, 57], unresponsiveness also to painful stimuli [44], recovery lagging awarded residency by months [40, 46], rare debuts after awarded residency [46], rare and subtle signs of immobilisation [34], staff suggesting at least some patients to be genuinely ill [64], patients treated long periods in hospital [44, 46], temporary symptom reversal upon benzodiazepine administration [35], and the conviction of many clinicians, doctors in particular [53, 55, 56] imply that feigned symptoms may not be the only explanation. Additionally, similar reaction patterns, including pervasive refusal syndrome [79, 105], and catatonia [22], although also aetiologically opaque and presumably lacking properties permitting a culture-bound analysis, advise caution. With these exceptions, the presentation, incompatible with known neurological and medical conditions, and susceptible to endemic spread, warrants, in addition to simulation, that the proposal of a functional disorder be taken into consideration.

## Concluding remarks

The regional distribution and selective affliction contradict trauma and stress as chief drivers of resignation syndrome [11, 53, 55, 57] and instead suggest features attributable of the Swedish context, and perhaps the afflicted groups, as aetiologically potent. Importantly, this analysis does not amount to a rejection of trauma and stress, nor of these having had any effect. On the contrary, behaviour within and beyond control has likely been provoked and earned characteristics from individual life experience of affected individuals. However, this does not suffice as explanation.

Resignation syndrome *qua* culture-bound is although not universally accepted well supported [1, 22, 34, 39, 76, 115, 122, 123]. Conceivably, malingering [62, 63], iatrogenic effects [76], and functional symptoms attaining endemic properties in relation to sanctioned illness narratives [22] are proposals compatible with a generous interpretation of *culture-bound* provided the accommodation also of feigned illness is permitted. However, regardless of the details here presented, data does not suffice for the contribution of each alternative to be determined. An outstanding question, to be further explored in a coming analysis, is how mechanisms underlying an aetiology distinct from feigned illness, presumably a functional type disorder, can be conceived and invoked to explain the class of phenomena to which resignation syndrome belongs. Considering the repeated challenges this class poses to medicine and society, its examination is imperative.

Here however, the discussion will be limited to a few remarks pertaining to features of the resignation syndrome endemic which relate to constructivism. Apart from the obvious characteristics–stakeholders endorsing a narrative attaining official recognition through a medical diagnosis, and the employment of an incentivising medicolegal practice–extensively discussed above, other, more subtle, observations can be made.

It has been argued that some medical conditions are embedded with cultural meanings not directly derived from their nature, but which nevertheless shape how society responds to those afflicted and how those afflicted experience their condition [18]. Thus, some conditions are stigmatised (leprosy, HIV/AIDS, sexually transmitted disease), and some are contested (fibromyalgia, chronic fatigue syndrome, gender dysphoria). Resignation syndrome was undoubtedly contested and presumably this was due to specific cultural meanings.

Victimhood was awarded to an extent arguably exceeding that of ordinary medial conditions, even considering the trauma presumed or asserted, and not always in keeping with the dispassionate attitude expected from medical professionals. Alleged trauma and breach of human as well as asylum seeking rights were emphasised in reports from NGOs working to promote human rights for asylum seekers [43, 124]. The Swedish Paediatric Working Group for Refugee Children was arguably biased towards the trauma and stress understanding and adopted a similar position [56]. Certainly, victimhood was applicable to the resignation syndrome patients. However, on what merit appears less clear than what proponents argued at the time. Serving to curate a different cultural meaning, involving deceit and even crime, were allegations of illness staged for the purpose of attaining permanent residency [62, 63], in which case an altogether different kind of victimhood, owed to use and abuse of children, is implied.

These opposing meanings could partially be derived from the nature of resignation syndrome if the aetiology indeed incorporated trauma and stress as well as simulation. However, the point is that–setting aside exposed cases of simulation the generalisability of which is uncertain–available evidence warrants these views status merely as hypotheses in accounting for the full volume of affected children. From this perspective, the understandings of resignation syndrome patients as victims, criminals, or victims of crime, it may be argued, were results of construction integral to the practice of medicine and science immersed in the particular sociocultural context and sentiments of Sweden at the time. That these attributions resulted from construction does by no means preclude that they are correct to greater or lesser extent, individually or all together–in fact some such combination is plausible. However, recognising their preliminary nature, the notion of a *doxogenic illness* caused by a belief system cultivated by therapists and the media [15], comes to mind.

Cultural meanings have been argued to affect policy and management. In HIV/AIDS and epilepsy, stigma, it has been suggested, could limit the propensity to seek health care with negative effects on treatment and management [18]. In resignation syndrome, the effects of cultural meanings on policy and management were conceivably substantial. Relying on victimhood–in the sense of having lived through trauma and suffering from stress–the illness narrative not only drew attention away from alternative explanations, including feigned illness, but also encouraged residency permit as a medical intervention, a strategy marshalled by healthcare professionals and others. Society’s response–most importantly the medicolegal practice–was thus a consequence of the victimisation it had taken part in constructing, presumably to satisfy also needs exceeding the protection and accommodation of individual asylum seekers, including securing political and professional influence by demonstration of ideological and moral stance. And crucially society’s response was what made and later unmade, resignation syndrome.

Another aspect of constructivism with bearing on resignation syndrome is the proposal of illness experience as constructed by the individual acting in and toward the world. This perspective has received attention and support also in social and cultural branches of cognitive neuroscience [125–127] encompassing analyses of resignation syndrome [22, 122]. It incorporates not only illness experience but, through enactment, also illness behaviour. According to these models, manifestations of illbeing will acquire features enabling them to serve a purpose to those afflicted but also to those of that context. In this way illness becomes contingent on sociocultural processes, encompassing also scientific trends, in which individual and context act on purpose and instinct alike to promote interests nested on different levels of abstraction. An analysis of resignation syndrome elaborates this interdependency [22] and trends in symptom expression can be analysed thus.

Moreover, illness experience arising from enactment is likely to impact treatment response as embedded expectations pertaining to the understanding of the condition influence help-seeking, empowerment, and management. Relatedly, in functional symptoms, conviction of a biological cause has been noted to predict poor outcome [128], and in placebo research, the importance of expectations on physiological, cognitive, and behavioural parameters is well established [129, 130]. The endorsed understanding of resignation syndrome, and in particular the notion of recovery to be dependent on permanent residency, induces expectations which, when entertained by patient, the family, and healthcare professionals, could serve to maintain symptoms, in accordance with the enaction model, as long as residency remains an, although distant, yet possible outcome. The demonstration of positive outcome when refraining from invoking residency as a part of treatment while also preventing corresponding influence by separation from family and the ordinary treatment team, thus mitigating the negative effects expectations of recovery occurring only after residency presumably have, appears to support this interpretation [34].

The constructivist understanding, however, runs deeper than expectations affecting illness course by interfering with treatment. The worlds of patients with chronic illness have been observed to undergo dramatic changes following isolation. When family, social life, occupation, and free movement are barred, the foundation for a sense of self can be lost [18] and consequently, revaluation and construction of a novel illness congruent identity, advocacy, and peer group formation may ensue [14]. Such reorganisation of self and social worlds could have impacted resignation syndrome patients, their families, and others engaged, and may have served to curate auspicious conditions for the condition to endure in individual cases but also as a social phenomenon.

The circumstances suggested to have contributed to the rise and fall of resignation syndrome have now been recounted. Social and cultural cues served to construct an illness narrative and a medicolegal practice which, it was proposed, generated and maintained the endemic.

The question of whether the endemic could have been avoided is warranted, not to award blame but to prevent similar events from recurring. Scholarship on psychosomatic and transient mental illness [14, 15] identifies legitimisation, incentives, polarisation, conspicuousness, etcetera, to contribute, but also show how trends in illness expression develop organically and insidiously in sociocultural contexts. Nevertheless, it is difficult to reject the idea that had medicine been more attuned to sociocultural factors affecting illness behaviour and management, which presumably involves recognition of illness as something more than a natural kind [7], and thus an openness to the constructivist approach, a more appropriate response could have been marshalled and the endemic perhaps mitigated at an earlier stage.

Crucially, the medical profession is awarded the privilege of characterising illness and making diagnoses. Provided there is some truth to the constructivist notion in relation to trending illness, thus accepting the arguments here put forward, a diagnosis is not only a description of an entity but also a model affecting how illbeing is understood and expressed. Thus, symptoms, help-seeking, and practice adapt to diagnoses and thereby authorises them. By such *reification* [131], which can occur regardless of the validity of a diagnosis, iatrogenesis may result. Considering medicine’s first obligation–do no harm–this insight, deserves attention in teaching, practice, and research.

On that note, the resignation syndrome diagnosis was introduced for epidemiological purposes. In the light of what has been argued, *resignation* is misleading, *syndrome* mistakenly excludes non-medical stakeholders and levels of explanation, and the diagnosis has caused harm. With the endemic over, it has in any case served its epidemiological purposes and now obsolete, its revocation is warranted.

## Data Availability

No datasets were generated or analysed during the current study.
